# Bilateral Van Neck-Odelberg Disease: A Case Report

**DOI:** 10.7759/cureus.56676

**Published:** 2024-03-21

**Authors:** Syed Faisal Afaque, Praseeth K Radhakrishnan, Udit Agrawal, Vikas Verma, Suresh Chand

**Affiliations:** 1 Department of Pediatric Orthopedics, King George’s Medical University, Lucknow, IND

**Keywords:** van neck-odelberg disease, case report, pediatric, ischiopubic osteochondritis, bilateral

## Abstract

Van Neck-Odelberg disease, also known as ischiopubic osteochondritis, is a rare cause of buttock or groin pain in the pediatric age group. The challenge in its diagnosis is due to its radiologic similarity. Ischiopubic synchondrosis occurs in childhood and is seen before the fusion of the pubis and ischium. With the advancement of age toward skeletal maturity, ischiopubic synchondrosis reduces in size and gradually vanishes due to synostosis or bony union. Here, we report the case of a 13-year-old girl who came to our outpatient department with complaints of bilateral groin pain for one year. After a thorough evaluation, she was diagnosed with a case of bilateral Van Neck-Odelberg disease, or ischiopubic osteochondritis, and conservative management was planned. Closure of ischiopubic synchondrosis varies with age and is usually finished before puberty. In a typical scenario, such fusion of the pubis and ischium does not lead to any clinical symptoms. However, in a few instances, children may experience some pain in the groin, hip, or gluteal region, which results in restricted movements at the hip joint and can lead to limping while walking. Van Neck-Odelberg disease is rare in children, causing pain in the groin region. As it is a rare condition, diagnosis is often missed. The radiological appearance suggests many differential diagnoses, such as a stress fracture, neoplasm, or infection. Prompt diagnosis and treatment can relieve the symptoms.

## Introduction

Van Neck-Odelberg disease, also known as ischiopubic osteochondritis, is a rare cause of buttock or groin pain in the pediatric age group. Odelberg [1] and Van Neck [2] in 1923 and 1924, respectively, reported an enlargement of ischiopubic synchondroses (IPS) in symptomatic cases. They both described the symptoms accompanying the radiological appearance of IPS. This hyaline cartilage joint is seen between the ischium and the pubis and ossifies physiologically before puberty [3], thus predisposing children of this age to pain. Sometimes, mechanical stress can predispose to a painful condition, restricting the movement of the hip region. The primary challenge in its diagnosis is mainly because of its radiological similarity to pseudo-tumoral diseases and other pathological conditions, such as osteomyelitis, stress fractures, pathologic fractures, or post-traumatic osteolysis [4]. To our knowledge, multiple cases have been reported in the literature with unilateral complaints, but only a few with bilateral symptoms have been reported until now.

## Case presentation

**Figure 1:**
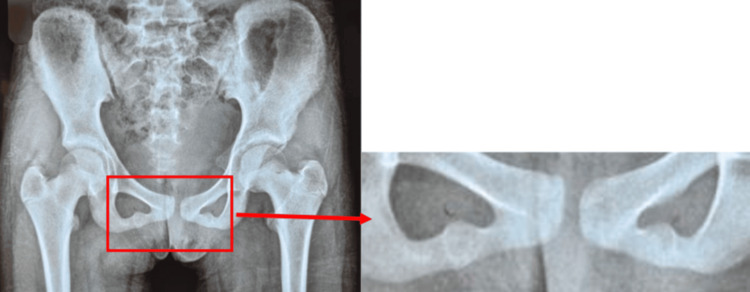
X-ray of the pelvis with both hips anteroposterior view showing bilateral ischiopubic synchondrondritis.

**Figure 2:**
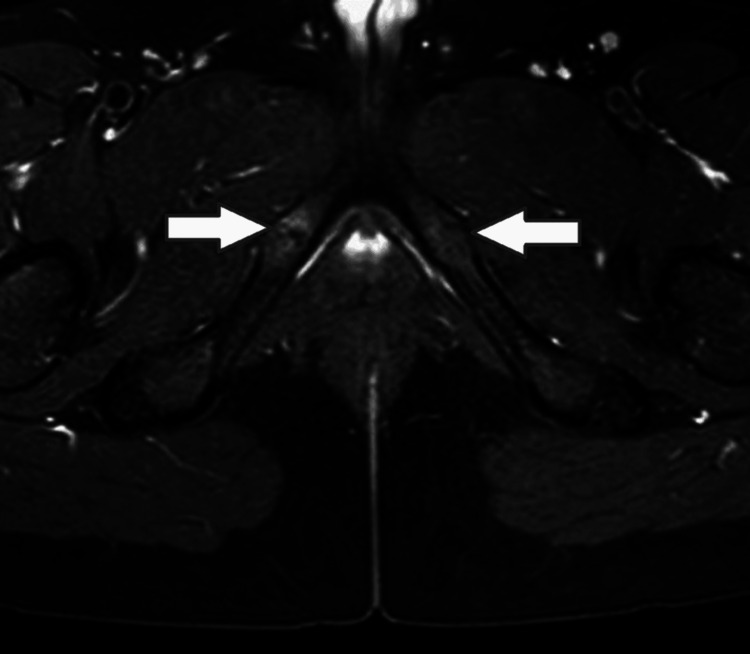
MRI showing an increased intensity with a fibrous band surrounded by an edematous change in the area of the bilateral ischiopubic synchondroses.

A 13-year-old girl reported to our outpatient department with complaints of bilateral groin pain for one year. The pain was a dull-aching type, which increased on activity and decreased on rest. The pain was non-radiating and not associated with any constitutional symptoms. There was no preceding trauma to the pain. The hip and spine appeared normal on clinical evaluation, and the patient had bilateral pubic bone tenderness. There was no local rise in temperature or any associated swelling. The total leucocyte count, erythrocyte sedimentation rate, and C-reactive protein were normal on routine serological evaluation. On radiologic evaluation, bilateral spherical radiolucent lesions were seen in the ischiopubic region, with no periosteal lesion or soft tissue swelling (Figure 1). MRI showed an increased intensity with a fibrous band surrounded by an edematous change in the area of the bilateral IPS, which was suggestive of bilateral Van Neck-Odelberg disease (Figure 2).

After a thorough evaluation, she was diagnosed with a case of Van Neck-Odelberg disease, or ischiopubic osteochondritis, and conservative management was planned. She underwent conservative treatment with non-steroidal anti-inflammatory medications (tablet naproxen 250 mg twice daily for three weeks and tablet serratiopeptidase 5 mg thrice daily for three weeks). She was advised to have strict bed rest for three weeks. Following this, the patient’s symptoms improved. The patient was initially followed up at four-week intervals for three months, followed by three monthly intervals for two years. No recurrence was noted on any follow-up visits.

## Discussion

Ischiopubic osteochondritis is a type of linkage between the inferior ischial and pubic rami, mainly composed of hyaline cartilage [3,5,6]. Ischiopubic synchondrosis occurs in childhood and is seen before the fusion of the pubis and ischium. With the advancement of the child’s age toward skeletal maturity, ischiopubic synchondrosis reduces in size and gradually vanishes due to synostosis or bony union [3,6]. The closure of ischiopubic synchondrosis varies with age and is usually finished before puberty. In a typical scenario, such fusion of the pubis and ischium does not lead to any clinical symptoms. However, in a few instances, children may experience some pain in the groin, hip, or gluteal region, which results in restricted movements at the hip joint and can lead to limping while walking [3,7-10]. These non-specific clinical features create difficulties in the differential diagnosis of equivocal findings on plain radiographs [3].

A radiolucent swelling in the prepubescent skeleton at the ischiopubic fusion zone was first described by Odelberg (1923) and Van Neck (1924) as “osteochondritis ischiopubica” [1-3]. This radiological finding is considered a normal ossification pattern [11] as the closure of the ischiopubic osteochondritis varies with age and can occur from 4 to 16 years old. These joints usually fuse or close asymmetrically rather than simultaneously. Therefore, an enlarged IPS is sometimes seen in older children before complete ossification [12]. As published in the literature, unbalanced mechanical stress, such as jumping or kicking, may result in an inflammatory reaction, which could delay the complete ossification of this synchondrosis, producing symptoms [3,6,7]. Its tumor-like appearance on conventional radiographs can be mistaken for a diagnosis of a stress fracture, tumor, or inflammation [3]. Radiological investigations such as X-ray and CT classically show increased size with sclerosis or lucency [3]. MRI is more sensitive and can help make early diagnoses [12]. Classically, there is an increase in signal intensity in T2 sequences with fatty saturation or short-tau inversion recovery due to bone edema at the site of synchondrosis, with evidence of cartilage and bone bridges inside [12]. It plays a vital role in ruling out a neoplasm, such as Ewing’s sarcoma, and increasing the diagnostic certainty [6,12]. Differential diagnosis can be difficult as it can resemble pubic rami stress fractures, which are one of the typical lesions and usually occur in athletes or after radiation therapy [3,6,13]. A stress fracture classically presents with hyperintense marrow edema on T2-weighted images and a hypointense irregular fracture line perpendicular to the long axis of the superior pubic ramus [3]. Tumors such as Ewing’s sarcoma ordinarily present with a pattern of permeative bone destruction with a soft tissue extension. Other differential diagnoses include post-traumatic osteolysis and osteomyelitis, where bone destruction and abnormal soft tissue shadows are seen [3,14], along with peripheral enhancement and abscess formation [3,15]. The treatment of Van Neck-Odelberg disease, or ischiopubic osteochondritis, is mainly conservative, with rest and supportive care [4].

## Conclusions

Van Neck-Odelberg disease is rare in children, causing pain in the groin region. As it is a rare condition, the diagnosis is often missed. The radiological appearance suggests many differential diagnoses, such as a stress fracture, neoplasm, or infection. Prompt diagnosis and treatment can relieve patients from their symptoms. Our patient had a rare case of bilateral Van Neck-Odelberg disease, which we could promptly diagnose and provide appropriate care.
